# Physiological Advantages of a Fiber-Enriched Plant-Based Dessert: Improved Glycemic Control and Appetite Regulation over a Commercial Dairy Product

**DOI:** 10.1021/acsomega.5c10700

**Published:** 2026-02-10

**Authors:** Maria Tereza Lucena Pereira, Andréa Cardoso de Aquino, Chiara Porro, Socorro Vanesca Frota Gaban

**Affiliations:** † Department of Food Engineering, Federal University of Ceara, Bloco 858, Campus do Pici, CEP, 60440-900 Fortaleza, Ceará, Brazil; ‡ Department of Clinical and Experimental Medicine, University of Foggia, 71121 Foggia, Italy

## Abstract

This study investigated the glycemic response and subjective appetite sensations elicited by a research prototype of a plant-based cocoa dessert, formulated with chickpeas, quinoa, sweet potatoes, and brown rice, and compared it with a commercial dairy dessert. Eleven participants (mean age = 27.0 ± 7.91 years; BMI = 24.11 ± 3.35 kg/m^2^) consumed portions containing 50 g of available carbohydrates in a randomized crossover design. Capillary blood glucose was measured at fasting and at 15–120 min postprandially. The incremental area under the curve (iAUC) was significantly lower for the plant-based dessert compared with the commercial dairy dessert (13003 ± 466 vs 13492 ± 492 mg·min/dL; *p* = 0.0021). At 15 min, postprandial glucose for the plant-based dessert was significantly lower than that for the dairy dessert (*p* < 0.05). Both desserts presented high glycemic index values (80.22 ± 44.00 vs 83.23 ± 46.78), but the plant-based dessert exhibited a lower glycemic load (16.60 vs 18.31). Subjective appetite analyses showed that the plant-based dessert produced significantly greater satiety at 90 and 120 min (*p* < 0.05) and higher fullness scores at 60, 90, and 120 min (*p* < 0.05) than the commercial dairy dessert. Conversely, hunger and prospective food consumption scores were significantly lower after the plant-based dessert at the same time points (*p* < 0.05). Appetite-specific sensations were also modulated, with significantly higher scores for salty and tasty hunger were observed after consumption of the plant-based dessert (*p* < 0.05). Both the plant-based and commercial dairy desserts exhibited inverse correlations between glycemia and hunger (*r* = −0.93, *p* = 0.0017; *r* = −0.92, *p* = 0.0032) and prospective food consumption (*r* = −0.96, *p* = 0.0004; *r* = −0.98, *p* < 0.0001), whereas satiety (*r* = 0.93, *p* = 0.0019; *r* = 0.95, *p* = 0.0007) and fullness (*r* = 0.91, *p* = 0.0009; *r* = 0.95, *p* = 0.0008) were positively correlated with glycemia, respectively. Per 100 g, the plant-based dessert had lower energy density, fat, and added sugars than the commercial dairy dessert. Overall, the plant-based cocoa dessert elicited a more favorable physiological profile, combining attenuated glycemic impact, moderate glycemic load, and enhanced satiety, supporting its potential as a nutritionally improved, functional alternative for the development of health-oriented dessert formulations.

## Graphical abstract

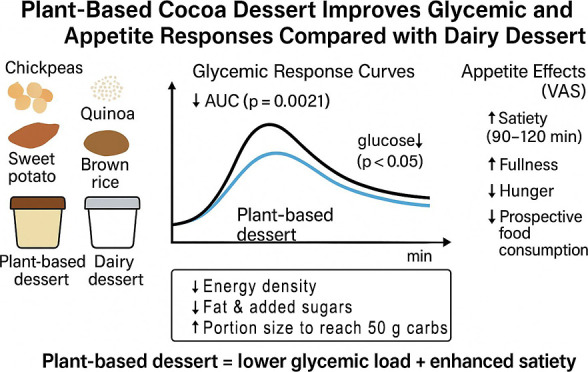

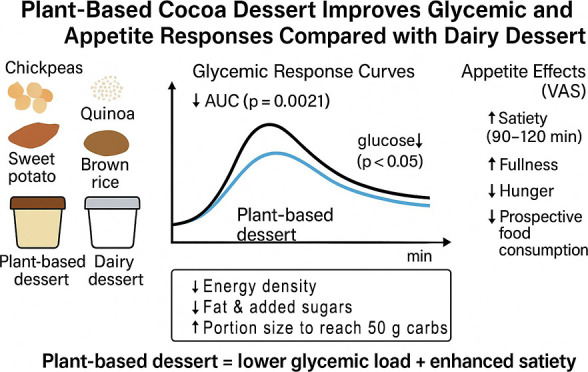

## 1 Introduction

Noncommunicable diseases (NCDs) remain the foremost cause of global mortality, largely driven by modifiable risk factors such as unhealthy diets and sedentary behavior.^1^ The escalating prevalence of obesity and metabolic disorders underscores the need for nutritional strategies that combine physiological efficacy with technological innovation. Diet quality, particularly the composition and structure of carbohydrate-rich foods, plays a decisive role in modulating postprandial glycemia, appetite control, and long-term metabolic health.^2^ In this regard, the development of functional foods that improve glycemic regulation while promoting satiety represents a pivotal direction in food science and nutrition.

The plant-based food sector has emerged as one of the most dynamic areas of nutritional innovation, offering sustainable and health-oriented alternatives to animal-derived products. Beyond their environmental advantages, plant-based formulations are recognized for their rich profiles of bioactive compounds, dietary fibers, and slowly digestible carbohydrates that contribute to attenuated glycemic responses and enhanced satiety.^3,4^ Such ingredients have demonstrated the capacity to modulate appetite-regulating hormones, reduce glucose absorption rates, and improve lipid and inflammatory biomarkers.^5,6^ Consequently, plant-derived ingredients not only address metabolic health concerns but also align with consumer demand for innovative foods with proven physiological functionality.

Recent advances in food technology have made it possible to reformulate traditional high-sugar, high-fat desserts, typically associated with unfavorable metabolic effects, into health-promoting alternatives without compromising sensory appeal. The incorporation of legumes, tubers, and pseudocereals into dessert matrices can enhance the techno-functional properties (texture, viscosity, stability) while simultaneously improving the nutritional and physiological quality of the product.^7^ From a biochemical perspective, the presence of soluble fibers, resistant starch, and polyphenols in such matrices may delay gastric emptying, reduce starch digestibility, and lower postprandial glucose and insulin peaks. These features position plant-based desserts as a promising innovation in the functional food market, capable of offering both hedonic satisfaction and metabolic benefits.^8^

From a technological standpoint, the structuring of plant-based desserts presents a unique challenge, requiring the optimization of hydrocolloid interactions, starch gelatinization, and protein network formation to ensure desirable rheological and sensory characteristics. The study by Lira et al.,^9^ conducted by our research group, demonstrated that the combination of chickpeas, quinoa, sweet potato, and rice in a cocoa dessert matrix produced a viscoelastic system following the Herschel–Bulkley model, with shear-thinning behavior indicative of a spoonable, stable, and homogeneous structure. The formulation exhibited improved water-holding capacity, consistent microstructure, and high sensory acceptance, confirming its feasibility from a technological and consumer perspective.

The present study represents a direct continuation of that work, expanding the investigation from the technological dimension to the functional validation of the same plant-based dessert. Specifically, this study aims to evaluate its metabolic performance by assessing glycemic response and subjective appetite sensations, thereby establishing a link between physicochemical functionality and physiological efficacy.

Several plant ingredients have demonstrated synergistic effects when used in functional food formulations. Chickpeas (*Cicer arietinum*) are rich in protein, soluble fiber, and bioactive peptides with documented antihypertensive, antioxidant, and antidiabetic properties.^10^ Rice (*Oryza sativa* L.) is a hypoallergenic cereal; however, when isolated and commercially processed, rice starch is predominantly classified as rapidly digestible due to its refined structure and high amylopectin content. In contrast, whole or minimally processed rice, when incorporated into complex food matrices, may contribute to a slower postprandial glycemic response.^11^ Quinoa (*Chenopodium quinoa*) contributes essential amino acids and phenolic compounds such as proanthocyanidins with anti-inflammatory and glucose-lowering activity.^12^ Sweet potato (*Ipomoea batatas*) provides complex carbohydrates and dietary fiber, and when cooked and cooled, it may retain small amounts of resistant starch that contribute to delayed glucose absorption and improved satiety.^13^ Finally, cocoa (*Theobroma cacao*) offers a unique combination of flavanols and methylxanthines associated with antioxidant, anti-inflammatory, and hypoglycemic effects.^14^ When strategically combined, these ingredients can produce a dessert matrix with both optimized sensory characteristics and measurable metabolic functionality.

Despite the growing interest in plant-based formulations, there remains limited evidence integrating technological, nutritional, and physiological parameters in a single food matrix. Understanding how formulation structure and composition influence both product stability and metabolic outcomes is crucial for developing next-generation functional desserts that contribute to dietary balance and metabolic wellness. The challenge lies in designing formulations that maintain palatability and texture while delivering measurable health benefits, bridging the gap between food engineering and nutritional science.

In this context, the present study validates the functional potential of a novel plant-based cocoa dessert previously optimized by our group.^9^ By integrating nutritional, physiological, and sensory assessments, it aims to provide a comprehensive understanding of how plant-based food design can influence postprandial metabolism and appetite regulation. The findings are expected to strengthen the technological and physiological foundation of this product, supporting the development of nutritionally optimized, consumer-accepted desserts that contribute to innovation in the plant-based functional food sector and promote metabolic health within sustainable dietary frameworks.

## 2 Materials and Methods

### 2.1 Participants and Recruitment

The study involved the deliberate selection of volunteers affiliated with the Federal University of Ceará, Pici Campus, including staff members as well as undergraduate and graduate students. Recruitment was conducted on campus between 8:00 am and 4:00 pm through face-to-face approaches and invitations sent via WhatsApp messages. Volunteers were invited to participate in a study aimed at evaluating glycemic response and the sensations of satiety and appetite following the ingestion of the tested products.

Participants were required to be at least 18 years of age, of either sex, and in good general health. Exclusion criteria included pregnancy, food allergies or intolerances (particularly to milk, lactose, or legumes), metabolic or gastrointestinal disorders, and the use of medications known to interfere with glucose metabolism. Health status was assessed through a pre-enrollment screening questionnaire covering chronic diseases, medication use, and food allergies or intolerances. Individuals reporting any of these conditions were excluded to prevent adverse reactions, as the test products contained sugar and dairy-derived ingredients. Eligible participants were instructed to maintain their usual dietary habits and physical activity levels throughout the study, to avoid alcohol and strenuous exercise for at least 24 h before each test session, and to observe a 10–12 h overnight fast prior to testing.

An initial pilot trial was conducted with 11 participants to assess feasibility, refine the study protocol, and standardize the test meals. In line with recommendations for pilot studies,^15,16^ the sample size was considered appropriate for exploratory purposes. Data from this phase served to optimize methodology and logistics but should be interpreted as preliminary findings requiring confirmation in larger trials.

### 2.2 Experimental Design

This pilot randomized crossover study included three treatments: (1) glucose solution (control), (2) creamy plant-based cocoa dessert, and (3) commercial dairy cocoa dessert (Figure 1). The study was designed to compare postprandial glycemic responses and subjective appetite sensations elicited by each formulation. The glycemic response to the meals was determined according to the FAO protocol.^17^ All test portions were standardized to provide 50 g of available carbohydrates.^17^

**Figure 1:**
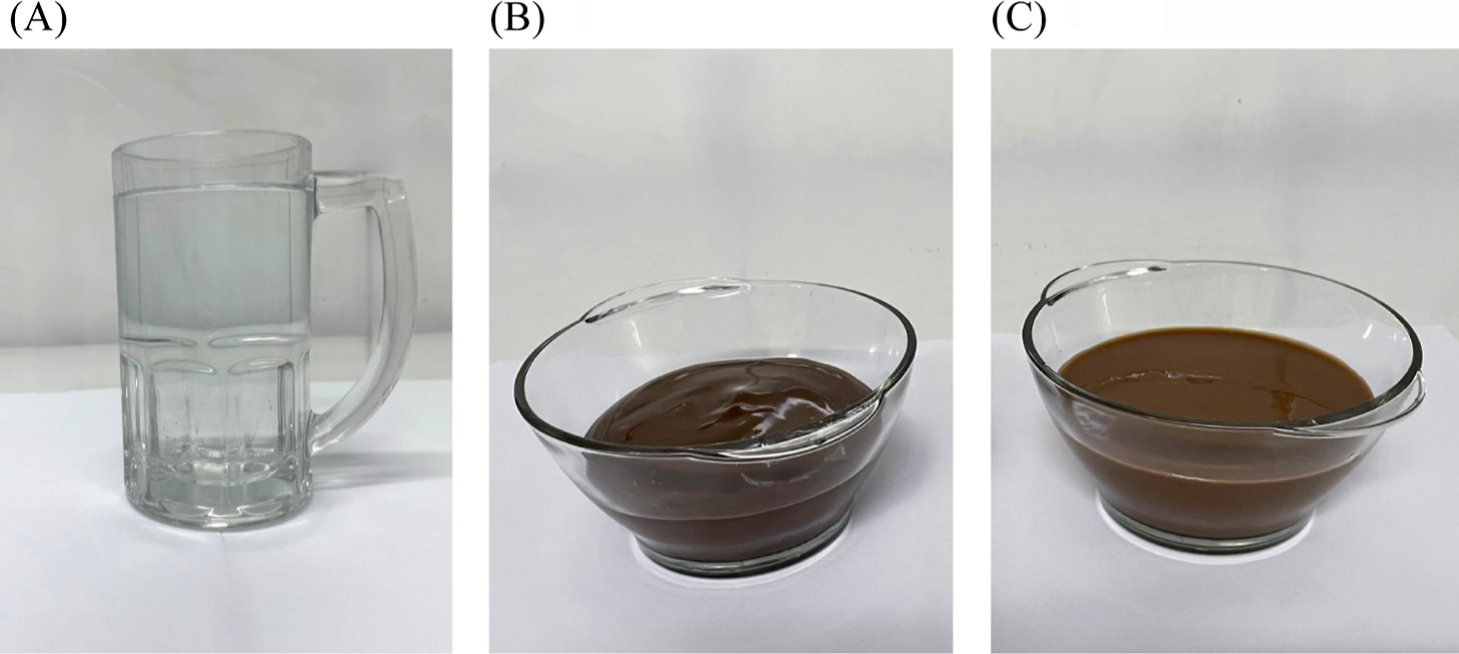
Test foods used in the glycemic response assessment: glucose solution (A), commercial dairy chocolate dessert (B), and plant-based cocoa dessert (C). All samples were standardized to provide 50 g of available carbohydrates. The images illustrate the visual characteristics of each product. Portion sizes corresponding to 50 g of available carbohydrates were: 250 g for the glucose solution, 241.66 g for the plant-based cocoa dessert, and 225 g for the commercial dairy chocolate dessert.

Participants attended six test visits conducted on nonconsecutive days, with a 2 day washout period between sessions to minimize carry-over effects. Each participant received all three treatments in a randomized order determined using a computer-generated random sequence (simple randomization). On the first and last test days, volunteers consumed the glucose drink containing 50 g of glucose dissolved in 250 g of water, while on the four intermediate test days, they consumed either the plant-based or the dairy cocoa dessert according to their assigned sequence. The overall experimental timeline followed a randomized crossover design with repeated measures, as illustrated in Figure 1. Each portion of glucose drink (250 g), plant-based cocoa dessert (241.66 g), or commercial dairy cocoa dessert (225 g) contained 50 g of available carbohydrates (Figure 2).

**Figure 2:**
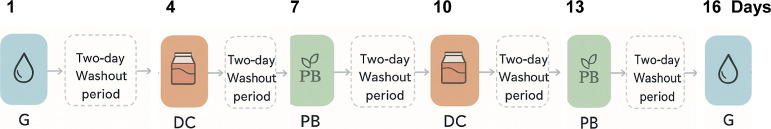
Randomized crossover study timeline over 16 days. Participants completed six test visits separated by 2 day washout periods: G (glucose solution; days 1 and 16); DC (dairy cocoa dessert; days 4 and 10); or PB (plant-based cocoa dessert; days 7 and 13).

Before each test session, participants were instructed to maintain their usual diet and physical activity throughout the study, to refrain from alcohol consumption and avoid strenuous physical activity for at least 24 h prior to testing, and to undergo a 10–12 h overnight fast before each session. These restrictions were implemented to ensure standardized metabolic conditions across all test visits.

### 2.3 Anthropometric Measurements

Previously, anthropometric measurements using standard methods, including height (with an accuracy of 0.1 cm) and weight (to the nearest 0.1 kg) were performed. Height was measured utilizing a standard stadiometer, ensuring that participants were not wearing shoes, and the measurement was recorded to the nearest centimeter. Weight was assessed using an electronic scale that underwent regular calibration, with results recorded to the nearest 100 g. During the weighing process, participants were instructed to wear light clothing without shoes, and no adjustments to the recorded weight were made for the clothing worn. Body mass index (BMI) was calculated using a person’s height and weight to designate a classification. The formula is BMI = kg/m^2^.^18^

### 2.4 Glycemic Response

Before the tests, subjects were instructed to minimize changes in their habitual diet and activity, abstain from alcohol consumption, and avoid strenuous physical activity during the study period. The tests took place after an overnight fast of 10 to 12 h. After the fasting period (time 0), a basal capillary blood sample was collected by finger prick using an automatic lancet holder with a lancet (Descarpack Plus). After the fasting collection (time 0), individuals consumed the reference or test foods for approximately 10–15 min. Additional blood samples from finger pricks were collected at 15, 30, 45, 60, 90, and 120 min after the start of feeding.^17,19,20^ Blood glucose readings were obtained using a glucometer (Accu-chek, Active, SP, Brazil).

### 2.5 The Glycemic Index and Glycemic Load

The glycemic index (GI) of the plant-based cocoa dessert and the commercial cocoa dairy dessert was calculated as the ratio between the incremental area under the blood glucose response curve (iAUC) for each dessert and the iAUC obtained after ingestion of the reference food (glucose), expressed as a percentage: GI = (iAUC_dessert/iAUC_glucose) × 100. The iAUC was calculated using the trapezoidal rule, with the fasting glucose concentration as the baseline. Glycemic load (GL) for each dessert portion was then calculated according to the standard definition: GL = (GI/100) × available carbohydrate (g per portion). The available carbohydrate content (g/portion) was obtained from the chemical composition of each dessert, considering the typical serving size used in the glycemic response tests.^21^

### 2.6 Subjective Feelings

Subjects were familiarized with these ratings before the commencement of the study (Figure 3). The anchors at 10 cm (to the right) and 0 cm (to the left) were used to evaluate each sensation (hunger, satiety, fullness, prospective food consumption). The 10 cm visual analogue scales (VAS) were printed on paper, and each response was measured using a digital caliper (Mitutoyo, Japan; precision 0.01 mm) to determine the distance from the left anchor to the participant’s mark, ensuring accuracy and reproducibility of the data.

**Figure 3:**
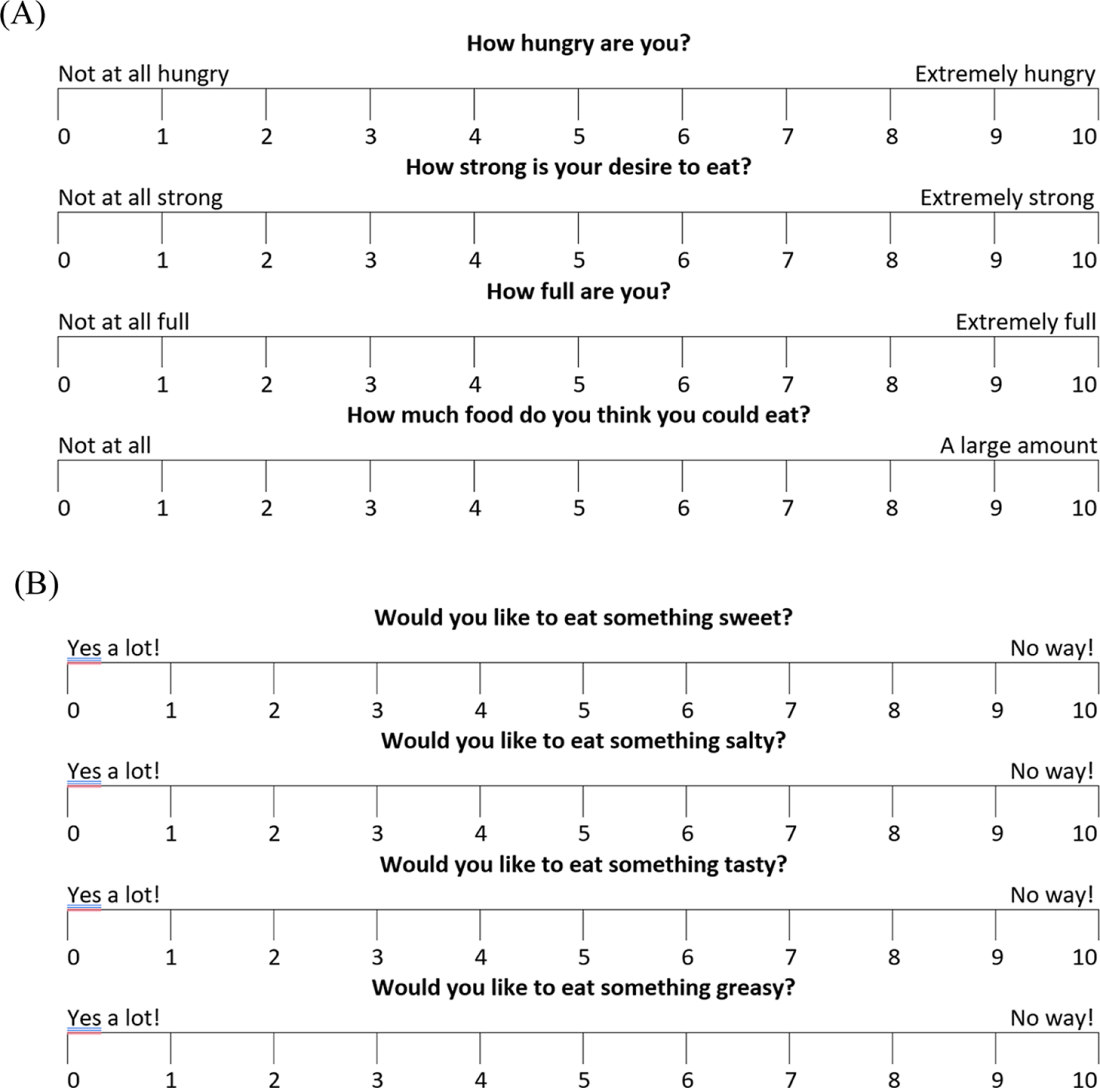
10 cm VAS to assess hunger, satiety, fullness, prospective food consumption (A) and sweet, salty, tasty and greasy hunger (B).

To determine hunger, subjects were asked: how hungry are you? With the answers from not at all hungry (0 cm) to extremely hungry (10 cm). How strong is your desire to eat? was rated from not at all hungry (0 cm) to extremely strong (10 cm). How full are you? was rated from not at all full (0 cm) to extremely full (10 cm). How much food do you think you could eat? was rated from not at all (0 cm) to a large amount (10 cm). Subjects were requested to make a vertical mark on each line that best matched how they were feeling at the time.

To determine sweet, salty, tasty and greasy hunger, subjects were asked: would you like to eat something sweet?; would you like to eat something salt?; would you like to eat something tasty?; and would you like to eat something greasy?, respectively, with the answer range from yes a lot (0 cm) to no way (10 cm). Each score was determined by measuring the distance from the left side of the line to the mark.^22,23^

The assessment involved concurrently evaluating satiety and appetite, along with the glycemic response, over 4 days during which participants consumed either the plant-based cocoa dessert or commercial dairy cocoa dessert, with two-day intervals between the assessments and alternating between the products.

The participant was given the following standard operating procedure (SOP) text, read verbatim: Please place a vertical mark on the horizontal line depending on how you feel now. You must rate how you feel now and not how you might think you should feel or how someone else might expect you to feel. Please consider the extreme labels, not at all and extremely, as the least and most hungry you have ever felt. When you complete the first question, please proceed to the next and continue through the questions until you have completed them all. It is worth noting that the SOP text was only read to the participant during the first occasion.^22,23^

### 2.7 Ethical Aspects

Before implementation, the study protocol was reviewed and approved by the Research Ethics Committee of the Federal University of Ceará (UFC/PROPESQ) (approval no. 006198/2022), in accordance with the ethical principles for research involving human subjects established by the Declaration of Helsinki and Brazilian regulations (National Health Council Resolution No. 466/2012). All participants were adequately informed, both orally and in writing, regarding the purpose, scope, procedures, and potential risks associated with the study. They were given the opportunity to ask questions and receive clarifications before agreeing to participate. Written informed consent was obtained prior to enrollment, with volunteers signing two copies of the Informed Consent Form (ICF), one retained by the participant and the other by the investigators. Only after signing the ICF were participants formally included in the study. They were explicitly informed of their right to withdraw from the study at any stage, without penalty or loss of benefits.

### 2.8 Raw Material

Chickpeas (*C. arietinum* L.) (Kicaldo Alimentos LTDA, SP, Brazil), quinoa (*C. quinoa*) (Excelência Selects Alimentos LTDA, CE, Brazil), sweet potatoes (*I. batatas*), brown rice (*O. sativa* L.) (Camil Alimentos LTDA, SP, Brazil), sugar (Parceria Alimentos LTDA, CE, Brazil), cocoa powder (*T. cacao*) (Mãe Terra Alimentos LTDA, SP, Brazil) and commercial dairy dessert (Danone LTDA, SP, Brazil) were purchased from local markets in Fortaleza, Ceará State, Brazil.

### 2.9 Plant-Based Cocoa Dessert Production

A creamy plant-based cocoa dessert was formulated following the procedure previously described by Lira et al.^9^ All ingredients were cooked exclusively in water, without the addition of salt or other components. Brown rice (*O. sativa* L.) was cooked in water at a 1:4 (w/v) ratio for 40 min. Chickpeas (*C. arietinum* L.) were first soaked in water (1:3, w/v) for 12 h under refrigeration (4 °C), after which the soaking water was discarded, and the grains were pressure-cooked in water at a 1:2 (w/v) ratio for 20 min. Quinoa (*C. quinoa*) was cooked separately in water at a 1:2 (w/v) ratio for approximately 30 min, while sweet potato (*I. batatas*) was pressure-cooked in water at a 1:3 (w/v) ratio for 20 min. Immediately after cooking, all ingredients were blended (Philco, PLQ1400, Brazil) with water for 20 min until a uniform and homogeneous mixture was obtained. Subsequently, sucrose and cocoa powder were gradually incorporated and mixed using a Philco PMX700 Red 700 W mixer for 5 min to ensure complete dispersion of dry components. The resulting dessert was portioned into sterilized glass jars, sealed, and stored under refrigeration (4–5 °C). For the preparation of 100 mL of plant-based cream, the specified amounts of each ingredient (chickpeas 15.39 g, quinoa 15.39 g, sweet potatoes 7.69 g, brown rice 3.85 g, sacarose 5 g, maltodextrin 5 g, and cocoa 3 g) were weighed (analytical balance, Ohaus, Adventure) and processed in a blender (Philco, PLQ1400, Brazil) with potable water, for approximately 20 min^9^

### 2.10 Proximate Composition

The nutritional value per 100 g and per portion of the plant-based and commercial dairy cocoa desserts consumed by volunteers was evaluated. The nutritional information for the commercial dairy dessert was sourced directly from the manufacturer. The creamy plant-based cocoa dessert was analyzed for macronutrient composition as follows: protein content was determined using the Kjeldahl method^24^ with a Kjeldahl distillation apparatus (TE-036/1, Tecnal Equipamentos para Laboratórios, SP, Brazil), lipid content was quantified according to the Bligh and Dyer method,^25^ moisture by gravimetric analysis by oven drying (SP Labor, SP, Brazil), ash by gravimetry after firing in a muffle furnace Q318A24 (Quimis, SP, Brazil), according to the Adolfo Lutz Institute,^26^ and total carbohydrate content was calculated by difference, subtracting the measured values of protein, lipids, ash, and moisture. The caloric value was estimated using the Atwater coefficients.^27^

### 2.11 Statistical Analysis

The results were expressed as mean ± standard error of the mean (SEM) and analyzed using Prism version 9.3.1 for Windows (GraphPad Software, CA, USA). The incremental area under the curve (iAUC) was calculated by using the trapezoidal rule with the negative values left out. Student’s *t*-test were used to assess whether the mean values of the groups were significant; a *p* value of <0.05 was considered significant.

The relationships between glycemic response and subjective measures (hunger, satiety, fullness, and prospective food consumption) were analyzed using Pearson correlation and linear regression models. The Pearson correlation coefficient (*r*) was used to evaluate the strength and direction of linear relationships between glycemic response (dependent variable) and the subjective measures (independent variables). Statistical significance was set at *p* < 0.05. Simple linear regression was performed to quantify the relationships between glycemic response (dependent variable) and each subjective measure (independent variables). The models provided regression equations (*y* = β0 + β1*x*), coefficients of determination (R2), and *p*-values to assess the significance of the slopes (β1). R2 values were used to explain the proportion of variance in glycemic response attributable to the independent variables. All data were checked for normality using visual inspection of residuals. Linear regression assumptions of homoscedasticity, linearity, and independence of errors were evaluated. Analyses were conducted separately for plant-based and commercial dairy desserts to allow for direct comparisons.

## 3 Results and Discussion

### 3.1 Study Population

The body mass index (BMI) is a well-established factor influencing glucose metabolism, as it reflects the balance between energy intake and expenditure and provides a simple yet effective indicator of nutritional status.^28,29^ In individuals within the eutrophic range (BMI 18.5–24.9 kg/m^2^), glucose metabolism tends to remain physiologically stable, with preserved insulin sensitivity and effective postprandial glucose clearance. This metabolic equilibrium contrasts with overweight or obese conditions, where increased adiposity is associated with insulin resistance, elevated fasting glucose, and impaired glucose tolerance.^30,31^

In this pilot study, which aimed to evaluate the glycemic response of healthy individuals after ingestion of the tested products, a total of 11 volunteers were recruited. The participants presented a mean body weight of 68.68 ± 12.28 kg and a BMI of 24.11 ± 3.35 kg/m^2^. These values fall within the eutrophic (normal) range according to WHO criteria, indicating that the study population was composed of normal-weight adults. This homogeneous anthropometric profile reduces variability related to metabolic status, minimizing potential confounding effects on postprandial glycemia.^28–31^ Therefore, differences in glycemic response can be confidently attributed to the experimental treatments rather than to disparities in BMI (Table 1).

**Table 1:** Age and Anthropometric Parameters of the Study Population

indicators	mean^a^± SD	min–max	median
age (years)	27.09 ± 7.91	19–43	24.00
body weight (kg)	68.68 ± 12.28	55–89	65.00
body height (m)	1.68 ± 0.06	1.56–1.75	1.67
body mass index (kg/m^2^)	24.11 ± 3.35	20.13–29.06	23.88

a Results are expressed as the mean and standard deviation (SD), (*n* = 11).

Similarly, Alptekin et al.^32^ conducted a short-term intervention with healthy female participants to assess the effects of milkshakes containing polydextrose and maltodextrin on appetite sensations, energy intake, and blood glucose. Their methodology, based on a sample of healthy, normal-weight individuals, parallels the present study design and supports the use of eutrophic participants to isolate the dietary effects on postprandial glycemic regulation.

### 3.2 Fasting and Postprandial Glycemic Responses

In healthy individuals, fasting blood glucose typically remains below 100 mg/dL, while concentrations above 126 mg/dL are indicative of diabetes.^33^ In the present study, the mean fasting glucose values of the 11 volunteers after 12 h of fasting were within the normal range across all test days: 95.00 ± 7.18 mg/dL (glucose solution), 95.73 ± 8.93 mg/dL (plant-based dessert), and 97.41 ± 8.29 mg/dL (commercial dairy dessert) (Figure 4A). These results confirm the absence of impaired fasting glucose and ensure the reliability of postprandial glycemic assessment.

**Figure 4:**
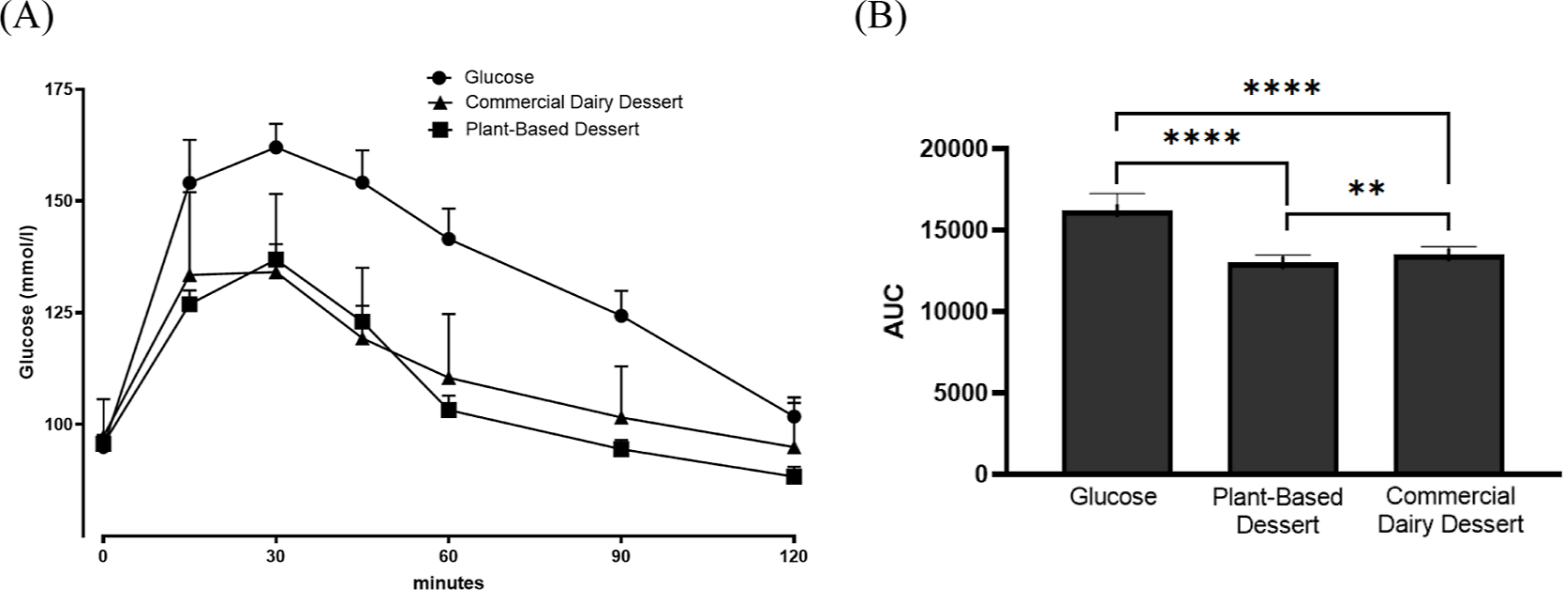
Glycemic response for glucose drink, commercial dairy dessert and plant-based dessert (A) and incremental area under the curve (iAUC) (B) for glucose drink, plant-based dessert and commercial dairy. Results are expressed as the mean and standard deviation. **P < 0.005; ****P < 0.0001.

Glycemic response (GR) reflects the postprandial variation in blood glucose concentration following the ingestion of carbohydrate-containing foods and is considered a key marker of metabolic health.^34^ It is influenced by multiple factors, including the rate of glucose absorption, the amount absorbed, and the efficiency of glucose clearance by tissues.^35^ Food composition and structure strongly modulate these processes, particularly the presence of dietary fiber, which delays gastric emptying and reduces glucose absorption.^36^ Our results (Table 2; Figure 4A) demonstrated that ingestion of 50 g of available carbohydrates produced distinct postprandial glycemic patterns depending on the food matrix. As expected, the glucose solution induced the highest glycemic excursion, peaking between 15 and 30 min (154.09 ± 44.98 and 162.04 ± 24.46 mg/dL, respectively), with levels gradually returning toward baseline at 120 min (101.82 ± 19.94 mg/dL).

**Table 2:** Postprandial Blood Glucose (mg/dL) after Consumption of Glucose Solution, Plant-Based and Commercial Dairy Desserts

	postprandial blood glucose (mg/dL)		
time (min)	glucose (*n* = 11)	plant-based dessert (*n* = 11)	commercial dairy dessert (*n* = 11)
0	95.00 ± 7.18^a^	95.73 ± 8.93^a^	97.41 ± 8.29^a^
15	154.09 ± 44.98^ac^	126.91 ± 14.64^b^	133.50 ± 18.47^c^
30	162.04 ± 24.46^a^	136.91 ± 16.22^b^	134.14 ± 17.45^b^
45	154.18 ± 33.74^a^	123.00 ± 16.74^b^	119.32 ± 15.74^b^
60	141.50 ± 31.75^a^	103.23 ± 15.14^b^	110.45 ± 14.26^b^
90	124.36 ± 26.17^a^	94.50 ± 9.36^b^	101.59 ± 11.40^c^
120	101.82 ± 19.94^a^	88.36 ± 10.21^b^	94.95 ± 9.91^c^

The commercial dairy dessert produced an intermediate response, with a rapid rise (peak: 134.14 ± 17.45 mg/dL at 30 min) and a delayed return toward baseline, maintaining elevated concentrations up to 90 min (101.59 ± 11.40 mg/dL). In contrast, the plant-based cocoa dessert elicited a significantly lower and more gradual postprandial response, peaking at 30 min (136.91 ± 16.22 mg/dL) and declining more rapidly thereafter, reaching the lowest values at 120 min (88.36 ± 10.21 mg/dL). At 15 min, blood glucose after the plant-based dessert (126.91 ± 14.64 mg/dL) was already significantly lower compared to the dairy dessert (133.50 ± 18.47 mg/dL; *p* < 0.05), suggesting delayed glucose absorption.

The sharper decline in blood glucose observed after consumption of the plant-based dessert, with values falling slightly below fasting levels at 90 and 120 min, is more plausibly attributed to acute differences in carbohydrate digestion and glucose absorption kinetics rather than to changes in insulin sensitivity. In a postprandial context, factors such as the structural complexity of the food matrix, the presence of slowly digestible or resistant carbohydrates, and delayed gastric emptying may modulate glucose appearance in circulation, contributing to a more rapid normalization of glycemia.^37^

The incremental area under the curve (iAUC, Figure 4B) confirmed these differences: glucose solution (16,209 ± 1052) > commercial dairy dessert (13,492 ± 492.2) > plant-based dessert (13,003 ± 466). Importantly, the glycemic response of the plant-based dessert was significantly lower than that of the dairy dessert (*p* = 0.0021).

From a clinical perspective, fasting and 2 h postprandial glycemia can be interpreted in light of the oral glucose tolerance test (OGTT), a diagnostic standard for diabetes.^38^ According to OGTT criteria, a 2 h postprandial value ≥ 200 mg/dL indicates diabetes. None of the test foods exceeded this threshold, confirming adequate glucose regulation among participants. Nonetheless, the kinetics of the curves differed markedly: the dairy dessert induced a faster and more sustained elevation, while the plant-based dessert yielded a slower, attenuated, and more favorable profile.

These results reinforce the concept that both the carbohydrate source and the overall food matrix influence postprandial glycemia.^39^ In the present study, although a statistically significant difference was detected between the two desserts, the absolute iAUC values were very similar, suggesting that the magnitude of this difference may have limited physiological impact. Therefore, factors other than dietary fiber, given that fiber content was not quantified, may have contributed to the slight reduction in glycemic response observed in the plant-based dessert. Previous studies have shown that food structure, ingredient interactions, and processing conditions can modulate glucose absorption independently of fiber content, partly explaining the modest differences found here.^40^

Overall, our findings indicate that the plant-based cocoa dessert elicits a postprandial glycemic response comparable to that of the commercial dairy dessert. While the small reduction in iAUC observed for the plant-based product may not be clinically substantial, it still supports its suitability as a viable alternative for individuals seeking plant-based or dairy-free options, including those with lactose intolerance or dietary restrictions. These results also contribute to the growing body of evidence that plant-based formulations can match the metabolic performance of traditional dairy products, while offering broader accessibility and potential functional benefits.

### 3.3 The GI and GL

The GI value determined for the plant-based dessert was 80.22 ± 44.00 (*n* = 11), whereas the commercial dairy dessert presented a GI of 83.23 ± 46.78 (*n* = 11) (Table 3). The GI is a well-established indicator that quantifies the rate at which carbohydrates in a given food are digested and absorbed, thereby influencing postprandial blood glucose concentrations. According to the standard classification proposed by Henry et al.,^41^ foods are categorized as low (≤55), medium (56–69), or high (≥70), with glucose serving as the reference food (GI = 100). The values observed in this study indicate that both desserts fall within the high glycemic index category, suggesting a rapid digestion and absorption of carbohydrates and, consequently, a marked increase in blood glucose levels.

**Table 3:** GI and GL of the Test Desserts

parameter	plant-based dessert (*n* = 11)	commercial dairy dessert (*n* = 11)	classification
GI	80.22 ± 44.00	83.23 ± 46.78	high GI (≥70)
GL	16.60	18.31	medium GL (11–19)

The slightly lower GI observed for the plant-based dessert compared with the commercial dairy formulation may be explained by differences in the available carbohydrate profile of the two products. In our formulation, the plant-based dessert contains predominantly complex carbohydrates derived from rice, chickpeas, quinoa, and sweet potato, which contribute mostly starch (%) and a smaller proportion of simple sugars (%). In contrast, the commercial dairy dessert provides a higher proportion of added sucrose/glucose-based simple sugars and a lower contribution from starch.

These compositional distinctions are relevant because starch-rich matrices, particularly those containing ungelatinized granules, resistant starch fractions, or viscous plant polysaccharides, tend to slow enzymatic hydrolysis and glucose release, thereby attenuating postprandial glycemia.^42^ In contrast, foods richer in rapidly available sugars typically elicit a faster glycemic response. Therefore, even though both products contained similar total carbohydrate amounts, the predominance of plant-derived starch in the plant-based dessert may have contributed to its modestly lower GI.

GL was used in this study as a complementary indicator of postprandial glycemic impact.^43^ In accordance with the procedures applied here, GL was calculated directly from the GI values obtained for each dessert and the measured amount of available carbohydrates in the portion administered during the glycemic response test. Thus, GL was computed using the standard equation: GL = (GI/100) × available carbohydrate (g per portion). Foods with a low GL (≤10) are considered to have a minimal glycemic effect, leading to a slow and modest increase in blood glucose. Those with a medium GL (11–19) cause a moderate elevation in blood glucose, while foods with a high GL (≥20) result in a rapid and significant rise in blood glucose concentration. This classification helps in dietary planning aimed at maintaining stable blood sugar levels and improving metabolic health. The GL value obtained for the plant-based dessert was 16.60 (*n* = 11), while that of the commercial dairy dessert was 18.31 (*n* = 11) (Table 4). Both values fall within the medium GL category (11–19), indicating a moderate glycemic impact. This suggests that consumption of either dessert produces a moderate postprandial increase in blood glucose concentration, rather than a rapid spike. The slightly lower GL observed for the plant-based formulation implies a more favorable glycemic response, which may be attributed to its higher fiber content and the presence of plant-derived bioactive compounds that can slow carbohydrate digestion and glucose absorption.

According to the World Health Organization, the GI and GL should not be regarded as the sole determinants of food quality or dietary adequacy.^2^ Although these parameters provide valuable insights into the postprandial glycemic response, a low GI or GL does not necessarily imply superior nutritional value or overall health benefits. The present study demonstrated that both the plant-based and dairy desserts exhibited high GI values and moderate GL values, suggesting a rapid yet quantitatively moderate impact on blood glucose. However, the physiological and metabolic effects of these foods must be interpreted within the broader context of their nutritional composition, including fiber content, lipid profile, and the presence of bioactive compounds.^2^ Indeed, evidence from randomized controlled trials indicates that reducing dietary GI or GL alone leads to minimal or no improvement in cardiometabolic risk markers. Consequently, the WHO highlights the importance of prioritizing dietary patterns rich in whole grains, dietary fiber, and high-quality carbohydrate sources, which contribute more effectively to metabolic health than focusing exclusively on glycemic metrics. For this reason, the WHO does not currently provide specific recommendations on dietary GI or GL values.^2^

Beyond glycemic parameters, the nutritional composition of the two desserts offers additional insight into their distinct metabolic responses. The plant-based dessert was formulated with ingredients such as brown rice (*O. sativa* L.), chickpeas (*C. arietinum* L.), quinoa (*C. quinoa*), and sweet potato (*I. batatas*), which are generally associated with more complex carbohydrate matrices and lower levels of free sugars compared with refined dairy-based formulations. Although dietary fiber was not directly quantified, the intrinsic compositional characteristics of these plant-based ingredients may have contributed to the attenuated glycemic response observed. In particular, the presence of plant-derived starches, resistant carbohydrates, and bioactive compounds, including polyphenols, may have slowed carbohydrate digestion and glucose absorption. In contrast, the higher proportion of rapidly available sugars and the more refined matrix of the dairy dessert may help explain its faster postprandial glucose rise. Collectively, these findings highlight that, beyond carbohydrate quantity and glycemic indices (GI and GL), the overall nutrient composition and food matrix play a central role in determining the physiological glycemic impact of foods.

### 3.4 Subjective Appetite Sensations

The VAS is a validated tool widely used to assess subjective appetite sensations in nutrition research.^23^ In this study, VAS was applied to monitor postprandial hunger and satiety at multiple time points, providing complementary information to glycemic response measurements.^22,44^ Although subjective, the method is reproducible under controlled conditions and allows detection of short-term changes in appetite related to food intake. Here, VAS outcomes were used solely to compare the satiating effects of the plant-based and dairy desserts following consumption.

After consumption, the commercial dairy dessert produced significantly higher scores for hunger and prospective food consumption at 60, 90, and 120 min compared with the plant-based dessert (*p* < 0.05) (Figure 5A,D). Conversely, the plant-based dessert generated significantly higher satiety scores at 90 and 120 min and fullness scores at 60, 90, and 120 min (*p* < 0.05) (Figure 5B,C). These results indicate that the plant-based formulation was more effective in sustaining satiety and reducing hunger, while the dairy dessert promoted a faster return of appetite and a greater desire for additional food.

**Figure 5:**
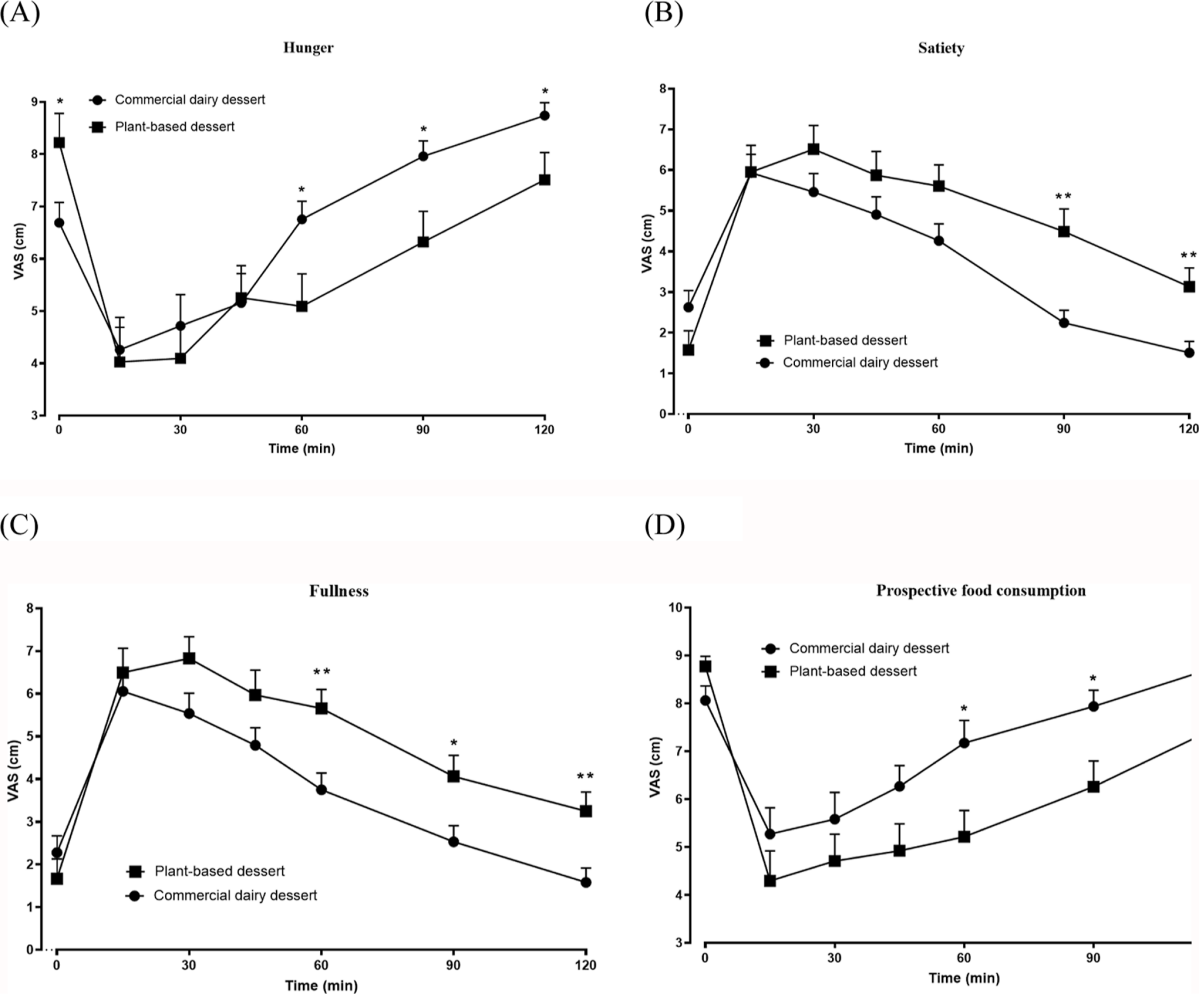
Variation of the hunger (A), satiety (B), fullness (C) and prospective food consumption (D) parameters at fasting (T0) and after consumption of commercial cocoa dairy dessert or plant-based cocoa desert (T15, T30, T45, T60, T90 and T120 min), in healthy volunteers (*n* = 11). Results are expressed as the mean and standard deviation.

Although dietary fiber was not quantified in this study, its potential postprandial effects cannot be ruled out. It is important to highlight that the differences in appetite responses may be closely related to the nature of the carbohydrate matrix and the structural characteristics of the plant-based ingredients, which are known to modulate gastric emptying, viscosity, and digestive kinetics. Previous studies have demonstrated that plant-derived food matrices, even when fiber content is not the primary differentiating factor, can enhance perceived satiety compared with more refined formulations.^45^ These findings support the notion that the intrinsic structure and compositional complexity of plant-based ingredients may have contributed to the more sustained satiety observed in the present study.

In the assessment of specific appetitive sensations, the VAS ranged from 0 (strong desire to eat) to 10 (no desire to eat) (Figure 6A–D). Higher mean scores were recorded for sweet and greasy hunger, while salty and tasty/savory hunger values were lower, indicating a typical morning preference for savory foods. Notably, higher scores for salty and tasty hunger were observed after consumption of the plant-based dessert (*p* < 0.05), suggesting a lower desire for additional savory foods. These findings demonstrate that the plant-based dessert not only enhances satiety but also modulates food cravings, promoting better appetite control and more balanced food choices.

**Figure 6:**
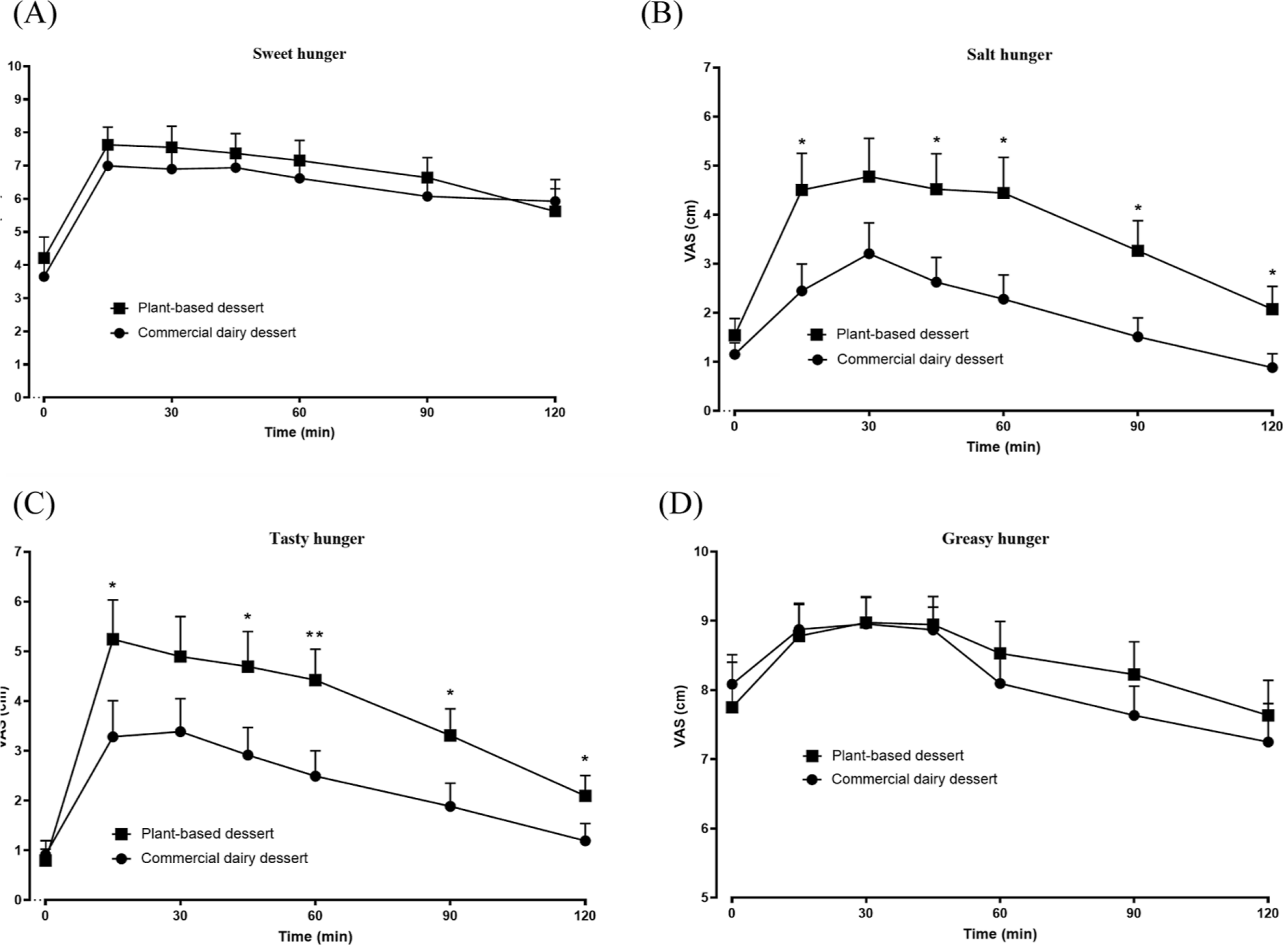
Variation of the sweet (A), salty (B), tasty (C) and greasy (D) hunger parameters at fasting (T0) and after consumption of commercial cocoa dairy dessert or plant-based cocoa desert (T15, T30, T45, T60, T90 and T120 min), in healthy volunteers (*n* = 11). Results are expressed as the mean and standard deviation.

Taken together, the results indicate that the plant-based dessert produced a slightly slower glycemic response and modestly higher satiety scores compared with the commercial dairy dessert. Although these differences were statistically significant, their magnitude was small and should be interpreted with caution. Nonetheless, the consistency between the glycemic and appetite-related outcomes suggests that the plant-based formulation may offer certain advantages within the specific conditions of this study.

From a product development perspective, these preliminary findings provide useful indications for refining plant-based dessert formulations, particularly regarding carbohydrate structure and ingredient interactions. However, broader conclusions about nutritional superiority or long-term metabolic benefits should not be drawn from these data alone, and further studies, including those assessing fiber content and larger sample sizes, are needed to confirm these observations.

### 3.5 Linear Regression and Pearson Correlation Analysis

Table 4 presents the linear regression equations and Pearson correlation coefficients between postprandial blood glucose concentrations and subjective appetite ratings for both desserts. For the plant-based dessert, negative and statistically significant correlations were observed between glycemia and hunger (*r* = – 0.93, *p* = 0.0017) and glycemia and prospective food consumption (*r* = – 0.96, *p* = 0.0004). These strong inverse relationships indicate that as blood glucose levels decreased over time, the sensation of hunger and the desire to eat increased correspondingly. In contrast, satiety (*r* = 0.93, *p* = 0.0019) and fullness (*r* = 0.91, *p* = 0.0009) were positively correlated with glycemia, suggesting that higher blood glucose concentrations were associated with greater perceived satiety. Similar, though weaker, positive correlations were also found for salty (*r* = 0.80, *p* = 0.0340) and greasy hunger (*r* = 0.79, *p* = 0.0430), indicating that higher glycemia may modestly reduce the desire for these specific food types. For the commercial dairy dessert, the same pattern was observed but with slightly higher correlation coefficients, confirming a consistent relationship between blood glucose and appetite control. Hunger (*r* = – 0.92, *p* = 0.0032) and prospective food consumption (*r* = – 0.98, *p* < 0.0001) were inversely correlated with glycemia, whereas satiety (*r* = 0.95, *p* = 0.0007) and fullness (*r* = 0.95, *p* = 0.0008) were positively correlated. These results reinforce that glycemic fluctuations strongly influence the dynamics of postprandial appetite sensations.

**Table 4:** Linear Regression and Pearson Correlation Analysis between Glycemia and Subjective Measures

subjective measure	regression equation	*R* ^2^	*r*	p-value
Plant-Based				
hunger	*Y* = −0,08381 × *X* + 15,53	0.88	–0.93	0.0017
satiety	*Y* = 0,08539 × *X* – 5527	0.87	0.93	0.0019
fullness	*Y* = 0,08775 × *X* – 5845	0.91	0.95	0.0007
prospective food consumption	*Y* = −0,06869 × *X* + 14,56	0.93	–0.96	0.0004
sweet	*Y* = 0,04868 × *X* + 1251	0.53	0.72	0.0635
salty	*Y* = 0,05658 × *X* – 2624	0.65	0.80	0.0274
savory	*Y* = 0,06947 × *X* – 3991	0.62	0.79	0.0340
greasy	*Y* = 0,02651 × *X* + 5494	0.82	0.90	0.0049
Commercial Dessert				
hunger	*Y* = −0,09470 × *X* + 17,03	0.84	–0.92	0.0032
satiety	*Y* = 0,1005 × *X* – 7508	0.91	0.95	0.0007
fullness	*Y* = 0,1039 × *X* – 7961	0.97	0.98	<0.0001
prospective food consumption	*Y* = −0,08080 × *X* + 16,15	0.97	–0.98	<0.0001
sweet	*Y* = 0,05015 × *X* + 0,4872	0.48	0.69	0.0838
salty	*Y* = 0,04765 × *X* – 3371	0.84	0.92	0.0033
savory	*Y* = 0,05761 × *X* – 4219	0.90	0.95	0.0009
greasy	*Y* = 0,03700 × *X* + 4069	0.81	0.90	0.0052

Overall, both formulations demonstrated significant associations between blood glucose levels and subjective appetite responses, confirming the physiological coupling between postprandial glycemia and perceived hunger or satiety. The slightly stronger correlations found for the dairy dessert may reflect its higher glycemic index, which produced sharper fluctuations in glucose and faster return of hunger. In contrast, the plant-based dessert exhibited more stable glycemic and appetite responses, consistent with its moderate GL and greater satiating potential.

### 3.6 Proximate Composition

The plant-based dessert and the commercial dairy dessert differed notably in their nutritional composition (Table 5). Per 100 g, the plant-based dessert provided 112.93 ± 1.86 kcal, while the commercial dairy dessert contained 142.30 kcal. When standardized to portions providing 50 g of total carbohydrates, the corresponding serving sizes were 241.66 g for the plant-based dessert and 225 g for the dairy dessert, yielding 272.91 and 320.18 kcal, respectively. Protein content was similar between products, although slightly higher in the dairy dessert (3.00 g/100 g) compared with the plant-based formulation (2.94 g/100 g). In contrast, fat content was markedly higher in the dairy dessert (4.70 g/100 g; 29.6% of energy) than in the plant-based product (2.01 g/100 g; 16.0% of energy). Added sugars also differed substantially, with the dairy dessert containing 14.00 g/100 g compared with 10.00 g/100 g in the plant-based dessert. Moisture and ash were only available for the plant-based dessert, with values of 73.92 ± 0.28 g/100 g and 0.44 ± 0.04 g/100 g, respectively.

**Table 5:** Nutritional Value per 100 g or per Portion Sizes Corresponding to 50 g of Available Carbohydrates for the Plant-Based Dessert (241.66 g) and the Dairy Chocolate Dessert (225 g)^a^

	plant-based dessert		commercial dairy dessert	
	100 g	241.66 g	100 g	225 g
energy (kcal/kg)	112.93 ± 1.86	241.66	142.30	320.18
protein (g/100 g; E %)	2.94 ± 0.06 (10.4%)	7.10 (10.4%)	3.00 (8.4%)	6.75 (8.4%)
total carbohydrates (g/100 g; E %)	20.69 ± 0.26 (73.3%)	50.00 (73.3%)	22.00 (61.5%)	49.50 (61.5%)
added sugars (g/100 g)	10.00	24.17	14.00	31.50
fat (g/100 g; E %)	2.01 ± 0.16 (16.0%)	4.68 (16.0%)	4.70 (29.6%) n.d.	10.58 (29.6%) n.d.
moisture (g/100 g)	73.92 ± 0.28	178.64 ± 0.68	n.d.	n.d.
ash (g/100 g)	0.44 ± 0.04	1.06 ± 0.10		

a E: energy. n.d., not determined (data not available on the commercial product label and not experimentally analyzed).

The observed differences in nutritional composition between the two desserts are relevant for interpreting their physiological effects. The plant-based dessert exhibited lower energy density and reduced fat and sugar content, which are nutritional characteristics typically associated with a more moderated postprandial metabolic impact.^46^ The larger portion size required to provide 50 g of carbohydrates reflects its lower carbohydrate concentration per gram, which may contribute to slower gastric emptying, greater volumetric satiety, and attenuated glycemic excursions.^47^

In contrast, the dairy dessert contained more fat and added sugars and had a higher energy density, factors known to influence both glycemic response and appetite regulation.^51^ The higher added sugar content may accelerate glucose absorption, whereas the higher fat content may delay gastric emptying but increase overall caloric intake.^52^ The absence of moisture and ash data for the dairy dessert limits detailed structural comparison, but the compositional differences reported are sufficient to explain the distinct glycemic and appetite profiles observed in the study.

Together, these findings highlight that macronutrient quality, energy density, and portion size all contribute to the postprandial response elicited by each dessert, supporting the interpretation that the plant-based formulation provides a nutritionally favorable profile compared with the commercial dairy product. Evidence from the literature supports this interpretation: quinoa-based diets have demonstrated reductions in postprandial glycemia in older adults with prediabetes, substitution of white rice for brown rice has been associated with improved glycemic markers in individuals with type 2 diabetes, and acute chickpea consumption has been shown to significantly reduce postprandial glucose iAUC in controlled trials.^48–50^ These findings reinforce the potential contribution of the plant-derived ingredients used in the formulation, chickpeas, quinoa, sweet potato, and brown rice, to the moderated glycemic response observed in this study.

## 4 Conclusion

In summary, the plant-based dessert elicited a slightly slower postprandial glycemic rise and modestly higher satiety ratings than the commercial dairy dessert. These physiological differences align with the distinct nutritional profiles of the products: the plant-based dessert exhibited lower energy density (112.93 kcal/100 g vs 142.30 kcal/100 g), lower fat content (2.01 g/100 g vs 4.70 g/100 g), and reduced added sugars (10.00 g/100 g vs 14.00 g/100 g), while requiring a larger portion to provide 50 g of carbohydrates (241.66 g vs 225 g). Although both desserts displayed high glycemic indices, the plant-based formulation demonstrated a moderate GL and a more gradual glycemic profile, which may be partially explained by differences in carbohydrate structure and matrix properties. This effect is likely related to the carbohydrate architecture arising from the plant-based dessert’s composition, which incorporates multiple ingredients naturally rich in fiber and complex carbohydrates. The appetite-related outcomes, although statistically significant, were small in magnitude and should be interpreted cautiously, particularly given that desserts are typically consumed at the end of a meal, when hunger is already reduced.

Collectively, these findings suggest that specific compositional attributes, particularly lower fat and added sugar content and reduced energy density, may contribute to the favorable short-term metabolic and appetite responses observed for the plant-based dessert under controlled conditions. However, the small effect sizes and the acute nature of the outcomes preclude broader conclusions regarding long-term nutritional or metabolic benefits. From a formulation perspective, the results offer preliminary guidance for optimizing plant-based dessert products with improved glycemic and satiety characteristics.

This pilot study has limitations, including the small sample size (*n* = 11), the inclusion of only healthy, normal-weight adults, and the absence of long-term metabolic markers such as insulin or incretins. Future research should include full compositional analyses, particularly dietary fiber quantification, and evaluate these formulations in real-meal contexts and longer-duration interventions. Studies in more diverse populations, including individuals with impaired glucose regulation, will be essential to validate these preliminary findings.

## References

[1] Dominguez L. J., Di Bella G., Veronese N., Barbagallo M. (2021). Impact of
Mediterranean diet on chronic non-communicable diseases and longevity. Nutrients.

[2] World Health Organization . Carbohydrate Intake for Adults and Children: WHO Guideline; World Health Organization: Geneva, 2023. 37490573

[3] Satija A., Hu F. B. (2018). Plant-based diets and cardiovascular
health. Trends Cardiovasc. Med..

[4] Springmann M., Wiebe K., Mason-D’Croz D., Sulser T. B., Rayner M., Scarborough P. (2018). Health and
nutritional aspects of sustainable diet
strategies and their association with environmental impacts: A global
modelling analysis. Lancet Planet. Health.

[5] Klementova M., Thieme L., Haluzik M., Pavlovicova R., Hill M., Pelikanova T., Kahleova H. (2019). A plant-based meal
increases gastrointestinal hormones and satiety more than an energy-
and macronutrient-matched processed-meat meal in T2D, obese, and healthy
men: A randomized crossover study. Nutrients.

[6] Wu S., Jia W., He H., Yin J., Xu H., He C., Zhang Q., Peng Y., Cheng R. (2023). A new dietary fiber
can enhance satiety and reduce postprandial blood glucose in healthy
adults: A randomized cross-over trial. Nutrients.

[7] Schmidt H. O., Oliveira V. R. (2023). Overview of the incorporation of legumes into new food
options: An approach on versatility, nutritional, technological, and
sensory quality. Foods.

[8] Hu Z., Xu Y., Xiong Y., Huang G. (2025). Mechanisms, functions, research methods
and applications of starch-polyphenol complexes in the synergistic
regulation of physiological parameters. Foods.

[9] Lira C. A. C., Viana J. D. R., Silva L. M. R., Gaban S. V. F. (2025). Sensory
acceptance
testing and rheological parameter analysis of a plant-based cocoa
dessert formulated with chickpeas, quinoa, sweet potatoes, and rice. Food Chem. Adv..

[10] Begum N., Khan Q. U., Liu L. G., Li W., Liu D., Haq I. U. (2023). Nutritional composition, health benefits,
and bioactive
compounds of chickpea (Cicer arietinum L.). Front. Nutr..

[11] Andriani R., Subroto T., Ishmayana S., Kurnia D. (2022). Enhancement methods
of antioxidant capacity in rice bran: A review. Foods.

[12] Agarwal A., Rizwana A. D., Tripathi T., Kumar K., Sharma K. P., Patel S. K. S. (2023). Nutritional and
functional new perspectives and potential
health benefits of quinoa and chia seeds. Antioxidants.

[13] Behera S., Chauhan V. B. S., Pati K., Bansode V., Nedunchezhiyan M., Verma A. K., Monalisa K., Naik P. K., Naik S. K. (2022). Biology
and biotechnological aspect of sweet potato (Ipomoea batatas L.):
A commercially important tuber crop. Planta.

[14] Tan T. Y. C., Lim X. Y., Yeo J. H. H., Lee S. W. H., Lai N. M. (2021). The health
effects of chocolate and cocoa: A systematic review. Nutrients.

[15] Montgomery R. (2025). Sample size
justification in feasibility studies: moving beyond published guidance. Pilot Feasibility Stud..

[16] O’Neill B. (2022). Sample size
determination with a pilot study. PLoS One.

[17] Brouns F., Björck I., Frayn K. N., Gibbs A. L., Lang V., Slama G., Wolever T. M. S. (2005). Glycaemic index methodology. Nutr. Res. Rev..

[18] Wei J., Liu X., Xue H., Wang Y., Shi Z. (2019). Comparisons of visceral
adiposity index, body shape index, body mass index and waist circumference
and their associations with diabetes mellitus in adults. Nutrients.

[19] Brand-Miller J., Hayne S., Petocz P., Colagiuri S. (2003). Low-glycemic
index diets in the management of diabetes: A meta-analysis of randomized
controlled trials. Diabetes Care.

[20] Rytz A., Adeline A., Lê K.-A., Tan D., Lamothe L., Roger O., Macé K. (2019). Predicting
glycemic index and glycemic
load from macronutrients to accelerate development of foods and beverages
with lower glucose responses. Nutrients.

[21] Jayedi A., Soltani S., Jenkins D., Sievenpiper J., Shab-Bidar S. (2022). Dietary glycemic index, glycemic load, and chronic
disease: An umbrella review of meta-analyses of prospective cohort
studies. Crit. Rev. Food Sci. Nutr..

[22] Blundell J. E., de Graaf C., Hulshof T., Jebb S. A., Livingstone B., Lluch A., Mela D. J., Salah S., Schuring E., van der Knaap H., Westerterp M. (2010). Appetite control: Methodological
aspects of the evaluation of foods. Obes. Rev..

[23] Flint A., Raben A., Blundell J. E., Astrup A. (2000). Reproducibility, power
and validity of visual analogue scales in assessment of appetite sensations
in single test meal studies. Int. J. Obes. Relat.
Metab. Disord..

[24] Kjeldahl J. (1883). Neue Methode
zur Bestimmung des Stickstoffs in organischen Körpern. Z. Anal. Chem..

[25] Bligh E. G., Dyer W. J. (1959). A rapid method of total lipid extraction and purification. Can. J. Biochem. Physiol..

[26] Adolfo Lutz Institute Analytical Standards of IAL: Chemical and Physical Methods for Food Analysis; Adolfo Lutz Institute: São Paulo, 2008.

[27] Atwater, W. O. Principles of Nutrition and Nutritive Value of Food; U.S. Department of Agriculture Farmers’ Bulletin No. 142; Government Printing Office: Washington, DC, 1910.

[28] Hossain S., Chowdhury A. I., Sarwer M. N., Akter F., Mukta N. A. (2025). Association
between blood glucose and body mass index with dietary diversity and
physical activity: a cross-sectional study on marma tribes of Bandarban
in Bangladesh. Health Sci. Rep..

[29] Walsh E. I., Shaw J., Cherbuin N. (2018). Trajectories of BMI change impact
glucose and insulin metabolism. Nutr., Metab.
Cardiovasc. Dis..

[30] Blaak E. E., Antoine J. M., Benton D., Björck I., Bozzetto L., Brouns F., Diamant M., Dye L., Hulshof T., Holst J. J., Lamport D. J., Laville M., Lawton C. L., Meheust A., Nilson A., Normand S., Rivellese A. A., Theis S., Torekov S. S., Vinoy S. (2012). Impact of
postprandial glycaemia on health and prevention of disease. Obes. Rev..

[31] Khanna D., Peltzer C., Kahar P., Parmar M. S. (2022). Body mass index
(BMI): A screening tool analysis. Cureus.

[32] Alptekin İ. M., Erdoğan E., İşler A., Yanalak E. C., Çakiroğlu F. P., Aras S. (2022). Short-term
effects of milkshake containing Polydextrose and maltodextrin on subjective
feelings of appetite, energy intake, and blood glucose in healthy
females. Nutr. Food Sci..

[33] Shkembi B., Huppertz T. (2023). Glycemic responses of milk and plant-based
drinks:
Food matrix effects. Foods.

[34] Vega-López S., Venn B. J., Slavin J. L. (2018). Relevance of the glycemic index and
glycemic load for body weight, diabetes, and cardiovascular disease. Nutrients.

[35] Rozendaal Y. J., Maas A. H., van Pul C., Cottaar E. J., Haak H. R., Hilbers P. A., van Riel N. A. (2018). Model-based
analysis of postprandial
glycemic response dynamics for different types of food. Clin. Exp. Nutr..

[36] Giuntini E. B., Sardá F. A. H., Menezes E. W. (2022). The effects of soluble dietary fibers
on glycemic response: An overview and future perspectives. Foods.

[37] Fukagawa N. K., Anderson J. W., Hageman G., Young V. R., Minaker K. L. (1990). High-carbohydrate,
high-fiber diets increase peripheral insulin sensitivity in healthy
young and old adults. Am. J. Clin. Nutr..

[38] Phillips P. J. (2012). Oral glucose
tolerance testing. Aust. Fam. Physician.

[39] Wolever T. M., Bolognesi C. (1996). Source and
amount of carbohydrate affect postprandial
glucose and insulin in normal subjects. J. Nutr..

[40] De
Natale C., Annuzzi G., Bozzetto L., Mazzarella R., Costabile G., Ciano O., Riccardi G., Rivellese A. A. (2009). Effects
of a plant-based high-carbohydrate/high-fiber diet versus high-monounsaturated
fat/low-carbohydrate diet on postprandial lipids in type 2 diabetic
patients. Diabetes Care.

[41] Henry C. J., Quek R. Y. C., Kaur B., Shyam S., Singh H. K. G. (2021). A glycaemic
index compendium of non-western foods. Nutr.
Diabetes.

[42] Meng H., Matthan N. R., Ausman L. M., Lichtenstein A. H. (2017). Effect
of macronutrients and fiber on postprandial glycemic responses and
meal glycemic index and glycemic load value determinations. Am. J. Clin. Nutr..

[43] Yu Y. T., Fu Y. H., Chen Y. H., Fang Y. W., Tsai M. H. (2025). Effect
of dietary glycemic index on insulin resistance in adults without
diabetes mellitus: A systematic review and meta-analysis. Front. Nutr..

[44] Chambers L., McCrickerd K., Yeomans M. R. (2015). Optimising foods for satiety. Trends Food Sci. Technol..

[45] Muhlhausler B. S., Belobrajdic D., Wymond B., Benassi-Evans B. (2022). Assessing
the effect of plant-based mince on fullness and post-prandial satiety
in healthy male subjects. Nutrients.

[46] Hägele F. A., Herpich C., Koop J., Grübbel J., Dörner R., Fedde S., Götze O., Boirie Y., Müller M. J., Norman K., Bosy-Westphal A. (2025). Short-term
effects of high-protein, lower-carbohydrate ultra-processed foods
on human energy balance. Nat. Metab..

[47] Cisse F., Pletsch E. A., Erickson D. P., Chegeni M., Hayes A. M. R., Hamaker B. R. (2017). Preload of slowly
digestible carbohydrate microspheres
decreases gastric emptying rate of subsequent meal in humans. Nutr. Res..

[48] Tey S. L., Salleh N., Henry C. J., Forde C. G. (2018). Effects of consuming
preloads with different energy density and taste quality on energy
intake and postprandial blood glucose. Nutrients.

[49] Witek K., Wydra K., Filip M. (2022). A high-sugar diet consumption, metabolism
and health impacts with a focus on the development of substance use
disorder: a narrative review. Nutrients.

[50] Díaz-Rizzolo D. A., Acar-Denizli N., Kostov B., Roura E., Sisó-Almirall A., Delicado P., Gomis R. (2022). Glycaemia fluctuations improvement
in old-age prediabetic subjects consuming a quinoa-based diet: a pilot
study. Nutrients.

[51] Mitta J. R., Vanamali D. R., Gara H. K. (2022). Impact of consumption of brown rice
on glycaemic and lipid profile in type 2 diabetics. Int. J. Adv. Med..

[52] Mah E., Uffelman C. N., Blonquist T. M., Wang D. D., Rehm C. D., Goltz S. R., Chu Y. (2025). Chickpea attenuates
postprandial
blood glucose responses: a systematic review and meta-analysis. Nutr. J..

